# Alaska Rabies Summit, Anchorage, Alaska—December 11, 2006

**DOI:** 10.3201/eid1311.070604

**Published:** 2007-11

**Authors:** Louisa Castrodale, Ivan Kuzmin, Susan A. Nadin-Davis, Erich Follmann, Kami Kandola, Isaac Sobol, Eleni Galanis, Alex Wandeler

**Affiliations:** * Alaska Section of Epidemiology, Anchorage, Alaska, USA; **†Centers for Disease Control and Prevention, Atlanta, Georgia, USA**; **‡**Center of Expertise for Rabies, Ottawa, Ontario, Canada; **§University of Alaska-Fairbanks, Fairbanks, Alaska, USA**; **¶**Stanton Territorial Health Authority, Yellowknife, Northwest Territories, Canada; # Department of Health and Social Services, Iqaluit, Nunavut, Canada; ** British Columbia Centre for Disease Control, Vancouver, British Columbia, Canada

The Alaska Rabies Summit held in December 2006 in Anchorage, Alaska, brought together professionals from the United States and Canada to share and discuss rabies prevention, control, and research efforts in the Arctic. The summit featured 7 presentations followed by discussion sessions to address shared challenges and areas for future research and collaboration. Summit participants included personnel from Alaska Departments of Environmental Conservation and Fish & Game, Alaska Native health corporation’s Offices of Environmental Health, North Slope Borough Department of Wildlife, US Department of Agriculture Wildlife Services, and private veterinarians.

## Rabies in the United States

Ivan Kuzmin, US Centers for Disease Control and Prevention, began by describing national rabies epizootiology and the 2 major phylogenetic clades of arctic rabies virus isolates from northern and western Alaska. Interestingly, some of the 2006 Alaska isolates fell into a new distinct clade. The significance of this information for local rabies epizootiology and potential control strategies is not clear, but ongoing surveillance and characterization of isolates will be valuable in documenting trends or the emergence of new clades.

## Rabies in Canada

Alexander Wandeler, Canadian Food Inspection Agency, described laboratory capacity in Canada where statistics for the arctic regions are limited in areas where population is sparse. One of the 2 Alaska virus clades clusters closely with virus variants recovered from the Canadian north. Moreover, variants closely related to those found in Canada have been identified in neighboring Greenland, observations that illustrate the ease by which animal movements on frozen waters spread rabies between jurisdictions in the northern environment.

## Rabies in Alaska

Louisa Castrodale, Alaska Division of Public Health, described activities of the Alaska Sections of Epidemiology and Laboratories for rabies surveillance, prevention, and control. Each year, rabies activity is confirmed among fox populations in the northern and western coastal areas of Alaska. More surveillance data are desired from areas that border known fox rabies enzootic areas and areas in the southeast where rabies has been detected in 2 bats. Spillover of arctic fox rabies into dogs is not uncommon and a method for minimizing this in enzootic rural areas has been to certify nonveterinarians as lay vaccinators (LVs) to administer animal rabies vaccinations. Recruiting, training, and retaining LVs is an ongoing challenge because they receive no compensation and vaccination duties are additional to their regular employment.

## Rabies Research in Northern Alaska

Erich Follmann, University of Alaska-Fairbanks, described recent studies of satellite-linked radio telemetry demonstrating that arctic foxes in remote undeveloped areas move widely during winter months, whereas those inhabiting areas of extensive development limit their movements to the same areas year round. This altered behavior pattern has significant implications for rabies epizootiology and may actually make an oral rabies vaccination program feasible in certain well-defined areas. In northern and western Alaska, arctic and red foxes coexist in some rabies enzootic areas. Red foxes are bigger and have been recently documented as preying on smaller arctic foxes. Such changes in interspecific interactions could affect regional epizootiology.

## Overview of Rabies Surveillance in the Northwest Territories

Kami Kandola, Stanton Territorial Health Authority, described rabies prevention and control activities in the Northwest Territories (NWT) of Canada. In 1995, the NWT Government established a Lay Vaccinator (LV) program. This joint initiative by the health and wildlife departments ensured that rabies vaccinations were made available on an ongoing and local basis. Challenges of the program have included difficulty recruiting LVs due to lack of remuneration, storage and administration of vaccines due to extreme cold temperatures, switch to monovalent vaccine and decreased participation by dog-owners more interested in canine distemper vaccine, and cultural beliefs that vaccine may adversely affect agility of sled dogs.

## Nunavut and Rabies

Isaac Sobol, Nunavut Department of Health and Social Services (DHSS), described Nunavut, which spans 20% of the land mass of Canada; none of the 25 small communities are connected by road. Environmental Health Officers rely on local wildlife officers in the communities for direct contact in many incidents involving suspected rabies. As expected, rabies is enzootic in arctic foxes and there is a substantial and susceptible dog population in most communities. Similar to the NWT, the Nunavut DHSS funds an LV program that provides animal rabies vaccines.

## Rabies in British Columbia

Eleni Galanis, British Columbia Centre for Disease Control, described the epizootiology of rabies in British Columbia where bats are the wildlife reservoir and multiple bat rabies variants circulate throughout the bats in this province. An unusual spill-over event occurred in May–June 2004, where skunks in Stanley Park, Vancouver, tested positive for a bat variant of rabies.

## Summary

Rabies in the arctic regions of Alaska and Canada remains a serious challenge because of the mobility of the wildlife reservoir and the limited human population density and infrastructure. Large-scale eradication efforts of rabies in foxes do not appear feasible; however, more regionalized control could be achieved. Before such initiatives are even considered, we need to better understand the ecology of both red and arctic foxes, the molecular characteristics of virus variants and their possible effects on reservoir susceptibility, transmission and circulation patterns, and the limitations to use of oral rabies vaccine in foxes, among other issues. Bringing together a multidisciplinary group of experts represents an initial step toward furthering our knowledge about rabies in the Arctic and options for control.

A related issue is that of limiting the ability of domestic dogs to transmit the virus to humans. Improving programs to increase rabies vaccination of dogs and basic animal control measures to limit the illness and death related to dog bites represent immediate and formidable challenges for local authorities. Sharing these challenges and approaches to potential solutions will continue to be valuable for Alaska and northwestern Canada.

Finally, other arctic nations that are similarly affected by animal rabies were not represented at the summit. The International Conferences for Rabies in the Americas and the International Congresses on Circumpolar Health are exemplary models for collaboration on disease issues that cross international borders. We suggest that future endeavors addressing Arctic rabies issues would prove valuable to international rabies prevention and control efforts and studies of circumpolar health.

